# Changing interactions among persistent species as the major driver of seasonal turnover in plant-caterpillar interactions

**DOI:** 10.1371/journal.pone.0203164

**Published:** 2018-09-06

**Authors:** Cintia Lepesqueur, Scheila Scherrer, Marcos C. Vieira, Mário Almeida-Neto, Danielle M. Salcido, Lee A. Dyer, Ivone R. Diniz

**Affiliations:** 1 Departamento de Zoologia, Instituto de Ciências Biológicas, Universidade de Brasília, Brasília, DF, Brasil; 2 Programa de Pós-Graduação em Ecologia, Instituto de Ciências Biológicas, Universidade de Brasília, CEP, Brasília, DF, Brasil; 3 Sistema Colégio Militar do Brasil, Colégio Militar de Brasília, Brasília, DF, Brasil; 4 Departamento de Ecologia, Instituto de Ciências Biológicas, Universidade Federal de Goiás, CEP, Goiânia, GO, Brasil; 5 Biology Department, University of Nevada, Reno, Nevada, United States of America; Helmholtz Zentrum Munchen Deutsches Forschungszentrum fur Umwelt und Gesundheit, GERMANY

## Abstract

β diversity of herbivorous insects in the tropics is usually very high, and there is often strong dissimilarity in herbivore species composition across different spatial scales and different abiotic gradients. Similarly, turnover is high for trophic interactions between herbivorous insects and their host plants. Two factors have been proposed to explain temporal or spatial differences in trophic interactions: changes in species composition and temporal changes in the behavior of shared species. The goal of this study was to evaluate determinants of high β diversity of trophic interactions between lepidopteran caterpillars and their host plants across dry and rainy seasons and their transitions. Over the course of a year, interaction diversity data were collected from 275 temporary plots in Cerrado vegetation, comprising 257 species of caterpillars, 137 species of host plants and 503 different trophic interactions. All these diversity parameters varied across seasons. Species assemblages of caterpillars and plants were different among the four seasons, and there was a high turnover of interactions between the seasons. The high temporal β diversity of trophic interactions was mostly due to interaction rewiring between co-occurring species, as opposed to changes in species composition over time.

## Introduction

Plant-herbivore networks (PHNs) are sensitive to biotic and abiotic factors, such that a network encountered in any particular environment may be dramatically different from functionally similar networks found in other environments [1–2]. Similarly, PHNs can vary through time due to habitat disturbance and ecological succession [2]. Another potential driver of temporal variation in PHNs is the phenological response of species to seasonal climatic variation. In tropical savannas, for instance, marked rainfall seasonality influences plant-herbivore interactions by altering patterns of reproduction and production of leaves and antiherbivore defenses in plants. The resulting changes in host plant quality in turn create seasonal differences in the population dynamics of herbivores and in the composition of associated arthropod communities [3–8]. Furthermore, precipitation directly influences reproduction, emergence, and diapause of insects [9–10]. Therefore, not only the composition of active insects and their host plant species change due to seasonal climatic variation, but also the very identity of their interactions.

Interaction turnover among communities separated in space or time can be conceptually disentangled into two components [2]. First, communities may differ in their interaction networks because of differences in species composition, since potential interactions cannot be realized in communities where one or more of the interacting partners are absent. Second, interactions among sets of species shared by multiple communities may change, since not all potential interactions are realized in a given community, even if all interacting species are present. Some studies have demonstrated that, in tropical environments, trophic interactions exhibit high interaction turnover across space, and that one of the mechanisms contributing to this turnover is the high host specificity of consumers, such as herbivorous insects [1]. Other mechanisms include turnover of resource assemblages, such as plant species; turnover of other consumers; or changes in interacting species, such as predators, parasites, mutualists, or competitors [2, 11]. Regarding temporal interaction turnover, however, the relative contributions of changing species compositions and changing interactions among shared species are not clear.

In this study, we analyzed the effect of temporal changes in communities on interactions between caterpillars and their host plants in Cerrado vegetation in central Brazil. The Brazilian Cerrado biome comprises a mosaic of vegetation types subject to extreme variation in rainfall between dry and rainy seasons, which strongly influences distributions of plants and animals [12]. Specifically, we tested two main hypotheses: 1) There is high turnover of host-caterpillar interactions among the dry and rainy seasons and the intervening transition periods. 2) If interaction turnover is due to the substantive seasonal changes in important biotic and abiotic parameters, the changes in interaction networks should be largest when caterpillar species diversity peaks—in the transition from the rainy to the dry season; this is because species richness is usually the strongest predictor of interaction diversity in biotic networks [13]. Relevant predictions from these hypotheses are that: 1) β diversity across seasonal interaction networks will be positively correlated with turnover of plants and caterpillars across the seasons. 2) Interaction turnover among seasons is partly driven by differences in larval interactions with host plants, including increasing diet breadth, decreasing breadth or switching host plants. 3) β diversity of interactions will be greatest when changes in species diversity are greatest.

## Materials and methods

The study system included externally feeding Lepidoptera and their host plants across four distinct seasons (dry season, dry-rainy transition, rainy season, rainy-dry transition, as defined below) in preserved areas of Cerrado *sensu stricto* in the Federal District, central Brazil (15°44'43" S—16°00'11" S and 47°50'50" W—47° 59',02" W). We completed 20–24 temporary plots (10 m diameter) monthly from March 2010 to March 2011, with a total of 275 plots. Due to the fact that there were plots without caterpillars (which were excluded from analyses), the final number of plots used for network analyses was 219, and the (network analysis) plots per month varied by season as follows: rainy 58, rainy-dry 65, dry 60, and dry-rainy 36. The uneven samples for analyses presents problems, but rarefaction indicated that patterns of diversity per plot across seasons are robust. For each plot, we identified and counted individuals of all species of plants over 20 cm in height, except the grasses (Poaceae). Similarly, we recorded the number of individuals of each species of caterpillar, along with their respective host plants. The leaves of all plants in the plots were inspected carefully for caterpillars for about 5 minutes per plant. The frequency of individual plants with caterpillars per plot was calculated as well as bitrophic interaction richness, which is the number of unique caterpillar-plant associations [14].

All caterpillars found in the plots were collected and reared to adults in an ambient temperature laboratory (i.e., no humidity or temperature control). The caterpillars were kept in individual pots and fed with leaves of the same relative age as the leaves from which they were collected in the field. In the laboratory (and occasionally in the field) these caterpillars were photographed for morphological characterization and identification. Adult Lepidoptera were identified by taxonomic specialists and by comparison with specimens in reference collections. Vouchers of adult specimens were deposited in the Entomological Collection of the Department of Zoology at the University of Brasilia.

### Data analysis

Data were grouped into four sets of networks on the season of sampling. Based on total monthly precipitation, we defined four 3-month seasons as follows: 1) dry season—June to August; 2) dry-rainy transition—September to November; 3) rainy season—December to February; and 4) rainy-dry transition March to May. For each of these periods, we quantified the number of caterpillar species, host plants, and unique caterpillar-plant interactions.

To examine the dissimilarity of interactions between all six pairs of seasons, we quantified the following for each pair: 1) the total dissimilarity between the seasonal networks (β_WN_); 2) the dissimilarity due to shared species interacting differently (β_OS_); and 3) the dissimilarity that was due to a change in the composition of species of caterpillars or plants between seasons (β_ST_) [2]. These three measures of interaction dissimilarity across seasons were calculated using two indices: Sorensen, which is based on the presence or absence of species, and Bray-Curtis, which also takes species abundances into consideration.

We used a Bayesian hierarchical model to evaluate changes in interaction richness across seasons, with plots nested within seasons and uninformative priors. To estimate model parameters, we used a Markov chain Monte Carlo (MCMC) simulation where each step in the chain estimates the difference between seasonal richness values. We used a 2000 step burn-in period followed by a 10000-step MCMC to generate the posterior density distribution for the differences in interaction richness between each pair of seasons. We calculated posterior probabilities of the null hypotheses (no difference in interaction richness between each pair of seasons) by quantifying the frequency with which the difference was equal to or greater than zero in each step of the post-burn-in MCMC. For example, if interaction richness is different between the two seasons across 99% of the post-burn-in MCMC sample (e.g., rainy—dry > 0), we can conclude that the probability that interaction diversity does not change between these seasons is 0.01. Bayesian analyses were performed in SAS v.9.4 using PROC GENMOD. For ease of interpretation, box plots for the raw data (rather than posterior distributions) are displayed in the results.

## Results

**Fig 1:**
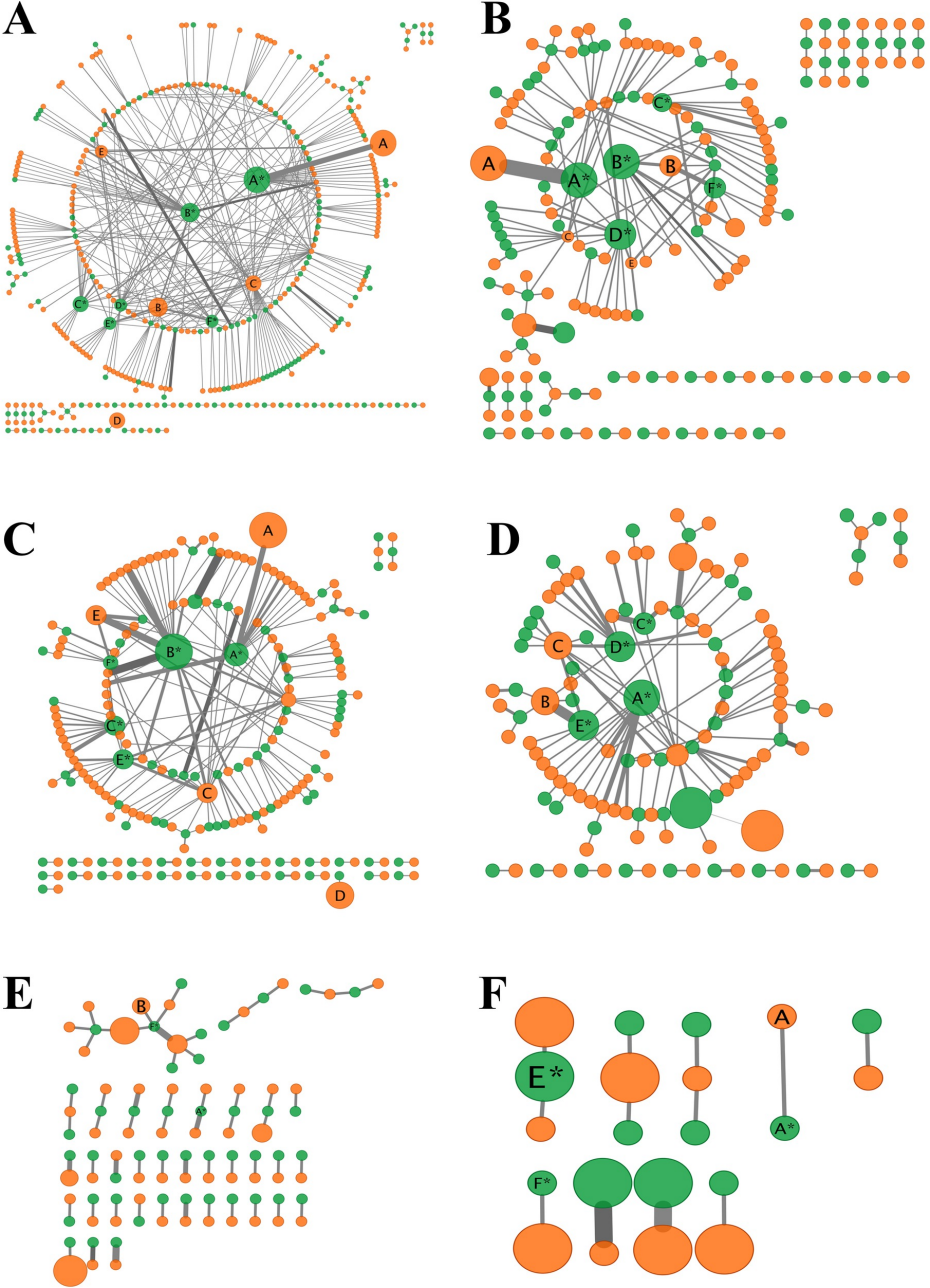
Network of interactions between plants and caterpillars at the study sites. (A) Full network, based on all interactions (similar to most networks assembled from data combined across time and space). Seasonal networks: (B) rainy season, (C) rainy to dry transition, (D) dry season, and (E) dry to rainy transition. All network parameters from the seasonal networks were different from each other and from the full network. A network at the smallest spatial scale (10 m diameter plot) is also depicted (F), since this was the unit that was used to calculate turnover. Edge sizes are based on interaction abundances and nodes sizes are scaled larger for the 5 most abundant species. The most abundant nodes (greater than 90th percentile) that are present in each network are labeled with species codes from the supplemental species lists (S2 Table). Caterpillar nodes (without asterisks) are denoted as: A: *Eomichla sp*. (Oecophoridae); B: *Pococera oeredella* (Pyralidae); C: *Inga phaeocrassa* (Oecophoridae); D: unknown species (undetermined family); E: *Compsolechia sp*. (Gelechiidae). Plant nodes (with asterisks) as: A: *Roupala montana* (Proteaceae); B: *Qualea multiflora* (Vochysiaceae); C: *Myrsine guianensis* (Primulaceae); D. *Miconia albicans* (Melastomataceae); E. *Acosmium dasycarpum* (Fabaceae); F. *Qualea parviflora* (Vochysiaceae).

We collected 257 species of caterpillars on 137 species of host plants, yielding a total of 503 different trophic interactions and four seasonal plant-caterpillar networks across the year (Fig 1). Species richness for caterpillars, host plants and trophic interactions varied over time (Table 1), and very few caterpillar species, plant species, and unique interactions were shared across all of the seasons. Exclusivity was high: 70% of all caterpillar species were exclusive to a single season, while 19.1% were shared by two seasons, 7.4% shared three seasons, and only 3.5% was found across all four seasons. The highest proportion of unique caterpillar species was found in the rainy-dry transition (Table 2). Only one species was shared between the dry season and the rainy-dry transition (Gelechiidae sp.), as well as the only one was shared by the rainy-dry and dry-rainy transitions (*Isognathus caricae* (Linnaeus, 1758)—Sphingidae).

**Table 1:** Total richness (mean/plot ±sd) of caterpillar species and host plants observed in each of four separate interaction networks based on the time factor in the cerrado *sensu stricto* of Brasilia (Distrito Federal), from March 2010 to March 2011.

Seasons*	Species richness of caterpillars (mean/plot ±sd)	Species richness of host plants (mean/plot ±sd)	Richness of interactions (mean/plot ±sd)
Dry season	75 (3.3±2.1)	49 (2.6±1.6)	129 (3.2±2.0)
Dry-rainy transition	50 (1.9±1.3)	44 (1.7±0.9)	58 (1.9±1.3)
Rainy season	107 (5.9±4.3)	76 (4.1±2.6)	178 (5.0±3.6)
Rainy-dry transition	139 (7.1±4.1)	81(4.2±2.0)	230 (5.7±3.3)
Total	257 (4.7±3.8)	137(3.3± 2.2)	503 (4,2±3,1)

* Dry season (June to August); dry-rainy transition (September to November); rainy season (December to February); rainy-dry transition (March to May).

**Table 2:** Number of unique species of caterpillars, host plants and interactions plus species that are shared across different seasonal networks of bitrophic interactions between caterpillars and host plants in the Cerrado *sensu stricto* of Brasilia (Distrito Federal); March 2010 to March 2011. Seasons separated by a hyphen (-) = transitions between seasons; (~) = Comparisons between seasons.

Seasons*	Caterpillar exclusive species	Host plant species	Number of interactions
Dry	25 (9.7%)	13 (9.5%)	80 (15.9%)
Dry-Rainy	23 (8.9%)	11 (8.0%)	39 (7.8%)
Rainy	54 (21.0%)	25 (18.2%)	136 (27.0%)
Rainy-Dry	78 (30.4%)	27 (19.7%)	172 (34.2%)
Subtotal exclusive species	180 (70.0%)	77 (55.5%)	427 (84.9%)
	Caterpillar shared species	Host plant species	Number of interactions
(Dry)~(Rainy)	7 (2.7%)	1 (0.7%)	9 (1.8%)
(Dry)~(Dry-Rainy)	1 (0.4%)	1 (0.7%)	2 (0.4%)
(Dry)~(Rainy-Dry)	18 (7.0%)	3 (2.2%)	26 (5.2%)
(Rainy)~(Dry-Rainy)	5 (1.9%)	4 (2.9%)	6 (1.2%)
(Rainy)~(Rainy-Dry)	17 (0.4%)	11 (8.0%)	16 (3.2%)
(Dry-Rainy)~(Rainy-Dry)	1 (0.4%)	4 (2.9%)	3 (0.6%)
(Dry~Rainy)~(Dry-Rainy)	3 (1.2%)	1 (0.7%)	0 (0.0%)
(Dry~Rainy)~(Rainy-Dry)	8 (3.1%)	13 (9.5%)	6 (1.2%)
(Dry)~(Dry-Rainy)~(Rainy-Dry)	4 (1.6%)	2 (1.5%)	3 (0.6%)
(Rainy)~(Dry-Rainy)~(Rainy-Dry)	4 (1.6%)	6 (4.4%)	1 (0.2%)
(Dry)~(Dry-Rainy)~(Rainy)~(Rainy-Dry)	9 (3.5%)	15 (10.9%)	4 (0.8%)
Subtotal shared species	77 (30.0%)	61 (44.5%)	76 (15.1%)
Total	257 (100%)	137 (100%)	503 (100%)

* Dry season (June to August); dry-rainy transition (September to November); rainy season (December to February); rainy-dry transition (March to May).

More than half of host plants (55%) were unique to only one of the four seasonal networks, and the highest proportion of unique species was found in the rainy-dry transition. The proportion of species shared by two, three and four seasonal networks was, respectively, 18%, 16% and 11% (Table 2). The largest proportion of unique caterpillar-plant interactions occurred at the rainy-dry transition (34%). The proportion of unique trophic interactions was also higher than those that were shared across seasons. Eighty-five percent of the interactions were unique to one of the seasons and only 15% were shared between two or more seasonal networks (Table 2).

The overall interaction dissimilarity (BWN) (Table 3) was high for both the Sorensen (ranging from 0.788–0.924) and the Bray-Curtis (ranging from 0.770–0.958) indices. In contrast, month-to-month BWN values within a season were substantially lower, ranging from 0–0.27, with mean BWN values within a season of 0.11. The largest interaction dissimilarities were found for dry-rainy transition versus the rainy-dry transition and the rainy season, respectively for Sorensen and Bray-Curtis Indices season (Bray-Curtis) (Table 3, S1 Table), and the smallest dissimilarities were found in the rainy-dry transition versus dry (Sorensen) and rainy (Bray-Curtis) (Table 3, S1 Table). Network dissimilarity across seasons was caused mainly by plant and caterpillar species shared across seasons interacting differently in different seasons (β_OS_, Table 3). This component was the largest contributor (75–81%) to the seasonal turnover (i.e. the total beta diversity) of interaction networks (Table 3). Differences in species composition (β_ST_) (Table 3) accounted for only 23–33% of total dissimilarity between the seasonal interactions networks.

**Table 3:** Sorensen indices calculated for plant-caterpillar interaction networks. BWN = total dissimilarity; BOS = dissimilarity due to shared species interacting differently; and BST = dissimilarity caused by differences in species composition. Dry = dry season; Dry-Rainy = transition between the dry and rainy seasons; Rainy = rainy season; Rainy-Dry = transition between the rainy and dry seasons.

	Sorensen Indices			
β_WN_	Rainy	Rainy-dry	Dry	Dry-rainy
	0.000			
Rainy-dry	0.868	0.000		
Dry	0.876	0.788	0.000	
Dry-rainy	0.906	0.924	0.904	0.000
β_OS_	Rainy	Rainy-dry	Dry	Dry-rainy
Rainy	0.000			
Rainy-dry	0.617	0.000		
Dry	0.600	0.512	0.000	
Dry-rainy	0.500	0.627	0.519	0.000
β_ST_	Rainy	Rainy-dry	Dry	Dry-rainy
Rainy	0.000			
Rainy-dry	0.251	0.000		
Dry	0.276	0.276	0.000	
Dry-rainy	0.406	0.297	0.313	0.000

**Fig 2:**
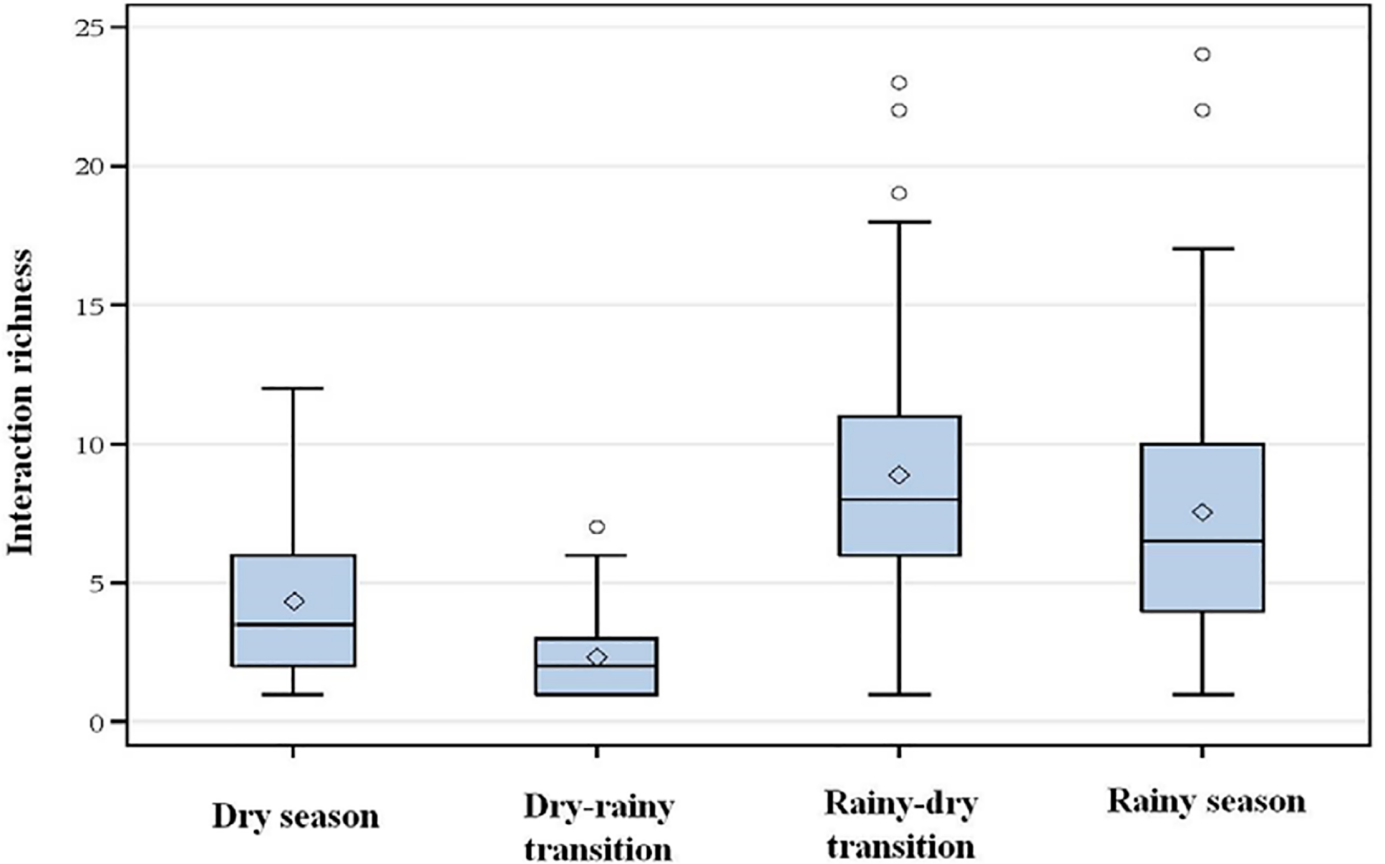
Mean plot-level interaction diversity (measured as richness) across four different seasons in the Cerrado. The rainy-dry transition plots were characterized by the highest interaction diversity and the dry-rainy transition exhibited the lowest interaction diversity.

Interaction richness per plot was significantly different for all paired comparisons among seasons. For all comparisons of interaction diversity across seasons, 95% HPD (highest posterior density) were greater than zero, (probability of no differences < 0.0001 for all comparisons). The greatest observed plot-level interaction richness was found in the rainy-dry transition (8.9 interactions per plot), followed by the rainy season (7.5 interactions per plot), and with much lower diversities in the dry season (4.3 interactions per plot) and the dry-rainy transition (2.3 interactions per plot) (Fig 2).

## Discussion

Despite the importance of understanding how species assemblages change in time and space, especially in dynamic and heterogeneous ecosystems, studies of interaction turnover are not common, and those that have carefully compared interaction diversity across time or space have yielded mixed interpretations of the determinants of turnover [2,11,15]. Interaction turnover can be decomposed into two components: the replacement of species between spatially or temporally different networks, and the replacement of interactions that occur between the species shared by those networks [2, 11]. In the current study, there was a high proportion of caterpillar species and their host plants that were unique to specific seasons within a year, but the turnover in small-scale networks (plots) was driven by high interaction turnover among shared species. Our results are limited to only one year, but the patterns are strong and fit predicted responses of interaction diversity to seasonal changes in vegetation.

A major hypothesis to explain β diversity of plant-insect interactions across different gradients and on different scales focuses on direct changes in the assemblages and numbers of host plant species and associated herbivores [11]. The Cerrado region where the study was conducted has a high degree of caterpillar species turnover across host plants, and this beta diversity increases with the phylogenetic distance between host plants [16]. This existing beta diversity across host plant species is part of what drives the seasonal turnover in caterpillar species, given that different plants have usable leaves accessible during different times of the year. However, even though most plant and caterpillar species occur in only one or two seasons, interaction β diversity was mostly determined by variable interactions among the relatively few species shared across seasons, not by differences in species composition. This apparent paradox suggests that the most abundant interactions occur within a core of species that persist for three or four seasons, and that the realized interactions among those relatively few species change in response to seasonality. The fact that the high-turnover of species contributes little to the total interaction β diversity suggests that many season-specific species are involved in relatively few peripheral interactions among themselves or with the core of more persistent species.

The period defined here as rainy-dry transition (March to May) is typically characterized by high caterpillar abundance and species richness, according to a number of studies in the Brazilian Cerrado, including longer-term studies up to 10 years in duration [17–21]. Our study corroborated these observations since over half of the caterpillar species occurred in this transition, and almost a third of these species were only found in the rainy-dry transition period. In contrast, the dry-rainy transition was characterized by the lowest caterpillar abundances and species richness, indicating that seasonal peak in production of new leaves in the Cerrado does not correspond to increases in herbivore or trophic interaction diversity [6,18].

In general, mechanisms that explain high dissimilarity of plant-herbivore networks across temporal or spatial gradients are not well studied, partly due to the problems inherent in studying insect-plant communities, for which natural history data are always incomplete [11, 14]. Problems with incomplete data were minimized in our study because we counted and identified all the individuals and species of caterpillars and plants, fully characterized plant caterpillar loads and caterpillar host relationships, and kept track of changes in species composition and differences in host plant use by caterpillars across our temporal gradient. It is likely that high interaction β diversity can be generated by shifts in multiple network parameters, such as specialization and connectance, across both spatial and temporal gradients, but it is better to examine this with fully characterized webs, which requires actual experimental evidence that interactions between species are consistent rather than casual observations of co-occurring species.

Analyses of food webs are critical because they generate measures that are relevant to functional diversity [13–14]. Because many species are involved in more than one trophic interaction, and these interactions can vary across space and time, food web analyses provide a more complete picture than a one-dimensional calculation of plant and caterpillar species diversity, [11,22]. For our bitrophic interaction networks, straight measures of species diversity provided relevant information about patterns of beta diversity over time—there was a high turnover of species in the rainy-dry transition compared to other seasons and low turnover in the dry-rainy transition compared to other seasons. However, the high interaction dissimilarity (Bray Curtis) over time provided the key insight into how changes in interactions can contribute to high beta diversity across a gradient. This change in interactions was most likely due to the tendency of caterpillar species to expand or decrease the number of host plants used in different habitats or different seasons, which affects both species diversity and interaction diversity at any one time or location. Caterpillars and their host plants respond to environmental changes in different ways, including changes in host plant quality, host leaf availability, lepidopteran oviposition preferences, caterpillar performance, and host shifting across caterpillar ontogeny [23–24].

In summary, over one year in the Brazilian Cerrado, we found clear differences in the composition of caterpillars and their host plants across four distinct seasons. There was a high turnover of interactions across these seasons. Despite extensive turnover of species across seasons, the high interaction β-diversity found for these networks was mostly due to changes in interactions among the restricted set of more persistent species.

## Supporting information

S1 Table Bray-Curtis indices calculated for plant-caterpillar interaction networks. (DOCX)

S2 Table Species codes and associated plant and caterpillar species or morphotypes. (DOCX)

S1 File R code for analyses. (R)

S2 File Data for analyses. (CSV)
