# Supplementary material for: Changing interactions among persistent species as the major driver of seasonal turnover in plant-caterpillar interactions
Source: PLoS One. 2018 Sep 6;13(9):e0203164. doi: 10.1371/journal.pone.0203164 (PMC6126867; doi:10.1371/journal.pone.0203164)
Supplement: S1 Table — (DOCX) [file pone.0203164.s001.docx]

**SUPPLEMENTAL MATERIAL FOR LEPESQUEUR *et* *al*: CHANGING INTERACTIONS AMONG PERSISTENT SPECIES AS THE MAJOR DRIVER OF SEASONAL TURNOVER IN PLANT-CATERPILLAR INTERACTIONS.**

**Table S1. Bray-Curtis indices calculated for plant-caterpillar interaction networks.** BWN = total dissimilarity; BOS = dissimilarity due to shared species interacting differently; and BST = dissimilarity caused by differences in species composition. Dry = dry season; Dry-Rainy = transition between the dry and rainy seasons; Rainy = rainy season; Rainy-Dry = transition between the rainy and dry seasons.

|  |  |  | **Bray-Curtis Indices** | | | |
| --- | --- | --- | --- | --- | --- | --- |
| β_WN_€ |  | β_WN_ € | Rainy | Rainy-dry | Dry | Dry-rainy |
|  |  |  | 0.000 |  |  |  |
| Rainy-dry |  | Rainy-dry | 0.770 | 0.000 |  |  |
| Dry |  | Dry | 0.939 | 0.835 | 0.000 |  |
| Dry-rainy |  | Dry-rainy | 0.958 | 0.957 | 0.937 | 0.000 |
|  |  |  |  |  |  |  |
| β_OS_ |  | β_OS_ | Rainy | Rainy-dry | Dry | Dry-rainy |
| Rainy |  | Rainy | 0.000 |  |  |  |
| Rainy-dry |  | Rainy-dry | 0.599 | 0.000 |  |  |
| Dry |  | Dry | 0.761 | 0.655 | 0.000 |  |
| Dry-rainy |  | Dry-rainy | 0.719 | 0.779 | 0.769 | 0.000 |
|  |  |  |  |  |  |  |
| β_ST_ |  | β_ST_ | Rainy | Rainy-dry | Dry | Dry-rainy |
| Rainy |  | Rainy | 0.000 |  |  |  |
| Rainy-dry |  | Rainy-dry | 0.171 | 0.000 |  |  |
| Dry |  | Dry | 0.178 | 0.180 | 0.000 |  |
| Dry-rainy |  | Dry-rainy | 0.239 | 0.178 | 0.168 | 0.000 |
