# Supplementary material for: Changing interactions among persistent species as the major driver of seasonal turnover in plant-caterpillar interactions
Source: PLoS One. 2018 Sep 6;13(9):e0203164. doi: 10.1371/journal.pone.0203164 (PMC6126867; doi:10.1371/journal.pone.0203164)
Supplement: S2 Table — (DOCX) [file pone.0203164.s002.docx]

| **Table S2. Species codes and associated plant and caterpillar species or morphotypes.** Plant species are shaded. Nodes in Fig 1 are labeled with the node codes. | | | |
| --- | --- | --- | --- |
| *Node_code* | *Species* | *Node_code* | *Species* |
| *P_1* | *Davila_elliptica* | *P_43* | *Erythroxylum_suberosum* |
| *P_2* | *Qualea_parviflora* | *P_44* | *Peixotoa_sp.* |
| *P_3* | *Caryocar_brasiliensis* | *P_45* | *Diplusodum_sp.* |
| *P_4* | *Byrsonima_pachyphylla* | *P_46* | *Erythroxylum_campestre* |
| *P_5* | *Machaerium_acutifolium* | *P_47* | *Asteraceae_sp.* |
| *P_6* | *Acosmium_dasycarpum* | *P_48* | *Myrtaceae_caule_quadrado* |
| *P_7* | *Roupala_montana* | *P_49* | *Croton_goyazensis* |
| *P_8* | *Maprounea_guianensis* | *P_50* | *Diospyros_burchellii* |
| *P_9* | *Miconia_albicans* | *P_51* | *Heteropteris_sp.* |
| *P_10* | *Qualea_grandiflora* | *P_52* | *Smilax_campestris* |
| *P_11* | *Stryphnodendron_adstringens* | *P_53* | *Cybianthus_detergens* |
| *P_12* | *Qualea_multiflora* | *P_54* | *Annona_coriacea* |
| *P_13* | *Miconia_ferruginata* | *P_55* | *Syagrus_flexuosa* |
| *P_14* | *Brosimum_gaudichaudii* | *P_56* | *Connarus_fulvus* |
| *P_15* | *Byrsonima_coccolobifolia* | *P_57* | *Planta_de_uma_folha_* |
| *P_16* | *Sp.1* | *P_58* | *Banisteriopsis_campestris* |
| *P_17* | *Erythroxylum_tortuosum* | *P_59* | *Himatanthus_obovatus* |
| *P_18* | *Sp.3* | *P_60* | *Austroplenckia_populnea* |
| *P_19* | *Sp.8* | *P_61* | *Mimosa_sp.* |
| *P_20* | *Baccharis_sp.* | *P_62* | *Serjania_sp.* |
| *P_21* | *Sp.9* | *P_63* | *Melastomataceae_sp.* |
| *P_22* | *Aspidosperma_macrocarpon* | *P_64* | *Desconhecida_sp.1* |
| *P_23* | *Fruto_ta√ßa* | *P_65* | *Mimosa_claussenii* |
| *P_24* | *Protium_ovatum* | *P_66* | *Alterna_espiculada* |
| *P_25* | *Kielmeyera_coriacea* | *P_67* | *Pterodon_pubescens* |
| *P_26* | *Byrsonima_verbascifolia* | *P_68* | *Eremanthus_glomerulatus* |
| *P_27* | *Lafoensia_pacari* | *P_69* | *Miconia_pohliana* |
| *P_28* | *Esenbeckia_pumila* | *P_70* | *Styrax_ferrugineus* |
| *P_29* | *Folha_pequena_com_inf.* | *P_71* | *Didymopanax_macrocarpum* |
| *P_30* | *Erythroxylum_deciduum* | *P_72* | *Annona_tomentosa* |
| *P_31* | *Nervura_saliente_abaxial* | *P_73* | *Jacquemontia_evovuloides* |
| *P_32* | *Ouratea_hexasperma* | *P_74* | *Ossaea_congestiflora* |
| *P_33* | *Bauhinia_sp._(folha_pequena)* | *P_75* | *Myrtaceae_desconhecida* |
| *P_34* | *Myrsine_guianensis* | *P_76* | *Dalbergia_miscolobium* |
| *P_35* | *Eremanthus_goyazensis* | *P_77* | *Sabicea_brasiliensis* |
| *P_36* | *Byrsonima_subterranea* | *P_78* | *Foto_1024200-02* |
| *P_37* | *Tocoyena_formosa* | *P_79* | *Machaerium_opacum* |
| *P_38* | *Sp.5* | *P_80* | *Odontadenia_lutea* |
| *P_39* | *Miconia_fallax* | *P_81* | *Andira_humilis* |
| *P_40* | *Rourea_induta* | *P_82* | *Symplocos_sp.* |
| *P_41* | *Vochysia_elliptica* | *P_83* | *Croton_antisyphiliticus* |
| *P_42* | *Psidium_pohlianum* | *P_84* | *Miconia_cuspidata* |
| *P_85* | *Psidium_sp.* | *P_128* | *Myrtaceae_moita* |
| *P_86* | *Pilosa_desconhecida* | *P_129* | *Periandra_sp.* |
| *P_87* | *Chamaecrista_adenophora* | *P_130* | *Kielmeyera_abdita* |
| *P_88* | *Peixotoa_goiana* | *P_131* | *Solanum_sp.* |
| *P_89* | *Pinta_preta* | *P_132* | *Byrsonima_sp.* |
| *P_90* | *Blepharocalyx_salicifolius* | *P_133* | *Hancornia_speciosa* |
| *P_91* | *Desconhecida* | *P_134* | *Siphoneugenia_densiflora* |
| *P_92* | *Gochnatia_polymorpha* | *P_135* | *Galactia_sp.* |
| *P_93* | *Phthirusa_ovata* | *P_136* | *Myrcia_linearifolia* |
| *P_94* | *Sclerolobium_paniculatum* | *P_137* | *leguminosa_peluda* |
| *P_95* | *Hyptis_lytroides* | *L_1* | *Picnotena_sp.* |
| *P_96* | *Palicourea_sp.* | *L_2* | *Edebessa_purens* |
| *P_97* | *Myrciaria_herbacea* | *L_3* | *Inga_haemataula* |
| *P_98* | *Redonda_tr√™s_folhas* | *L_4* | *Gonioterma_exquisita* |
| *P_99* | *Bauhinia_sp._(folha_grande)* | *L_5* | *Elachistidae_sp.1* |
| *P_100* | *Casearia_sylvestris* | *L_6* | *Dalcerina_tijucana* |
| *P_101* | *Piptocarpha_rotundifolia* | *L_7* | *Pococera_oeredella* |
| *P_102* | *Chresta_sphaerocephala* | *L_8* | *Eomichla_sp.* |
| *P_103* | *Banisteriopsis_sp.* | *L_9* | *Megalopyge_albicollis* |
| *P_104* | *Strychnos_sp.* | *L_10* | *Mazdacis_sp.* |
| *P_105* | *Rasteira* | *L_11* | *sem_id_(133)* |
| *P_106* | *Palicourea_rigida* | *L_12* | *Cicinnus_sp.* |
| *P_107* | *Tabernaemontana_affinis* | *L_13* | *Antaeotricha_subdulcis* |
| *P_108* | *Parece_cagaita* | *L_14* | *Inga_phaeocrassa* |
| *P_109* | *Annona_crassiflora* | *L_15* | *sp.2* |
| *P_110* | *Heteropteris_Trepadeira* | *L_16* | *Compsolechia_sp.1* |
| *P_111* | *Myrtaceae_de_borda* | *L_17* | *Inga_sp.* |
| *P_112* | *Aspidosperma_tomentosum* | *L_18* | *Elachistidae_sp.3* |
| *P_113* | *Periandra_mediterranea* | *L_19* | *sp.4* |
| *P_114* | *Pterandra_pyroidea* | *L_20* | *Cerconota_achatina* |
| *P_115* | *Calliandra_dysantha* | *L_21* | *Limacodidae_sp.* |
| *P_116* | *Palicourea_squarrosa* | *L_22* | *Oiketicus_kirby* |
| *P_117* | *Pavonia_sp.* | *L_23* | *Saturniidae_sp.9* |
| *P_118* | *Ocotea_sp.* | *L_24* | *Diaphania_sp.1* |
| *P_119* | *Pisonia_sp.* | *L_25* | *sp.9* |
| *P_120* | *Myrtaceae_folha_dura* | *L_26* | *sp.6* |
| *P_121* | *Desconhecida_sp.6* | *L_27* | *Mediavia_sp.* |
| *P_122* | *Bowdichia_virgilioides* | *L_28* | *Stenoma_sp.25* |
| *P_123* | *Tabebuia_ochracea* | *L_29* | *Phycitinae_sp.20* |
| *P_124* | *Salacia_crassiflora* | *L_30* | *Gelechiidae_sp.36* |
| *P_125* | *Myrtaceae_sp.1* | *L_31* | *Inga_oxybela* |
| *P_126* | *Dalechampia_caperonioides* | *L_32* | *Heraclides_thoas_brasiliensis* |
| *P_127* | *Anacardium_humile* | *L_33* | *sp.7* |
| *L_34* | *Phobetron_hipparchia* | *L_120* | *Phrudocentra_eccentrica* |
| *L_35* | *sp.8* | *L_121* | *Stenoma_hydraena* |
| *L_36* | *Aguna_albistria* | *L_122* | *Azamora_sp.* |
| *L_37* | *sp.10* | *L_123* | *Hemiceras_sp.* |
| *L_38* | *Psychidae_sp.* | *L_124* | *Gelechiidae_sp.81* |
| *L_39* | *Elbella_luteizona* | *L_125* | *sp.22* |
| *L_40* | *sp.11* | *L_126* | *Gelechiidae_sp.78* |
| *L_41* | *sp.12* | *L_127* | *Gelechiidae_sp.77* |
| *L_42* | *Tarema_sp.1* | *L_128* | *Antaeotricha_spurcatella* |
| *L_43* | *Thyrididae_sp.2* | *L_129* | *Antaeotricha_sp.5* |
| *L_44* | *Pseudasellodes_platygymma* | *L_130* | *Gelechiidae_sp.76* |
| *L_45* | *Druentica_rotundula* | *L_131* | *Inga_encamina* |
| *L_46* | *Deuterollyta_chrysoderas* | *L_132* | *Lethata_anophthalma* |
| *L_47* | *Incarcha_aporalis* | *L_133* | *Antaeotricha_sp.13* |
| *L_48* | *Gelechiidae_sp.9* | *L_134* | *Inga_ancorata* |
| *L_49* | *Diaphania_sp.* | *L_135* | *sp.23* |
| *L_50* | *Stenoma_muscula* | *L_136* | *Notodontidae_sp.1* |
| *L_51* | *Stenoma_cathosiota* | *L_137* | *Stenoma_sp.22* |
| *L_52* | *Inga_sp.13* | *L_138* | *Stenoma_straudingerana* |
| *L_53* | *Antaeotricha_sp.21* | *L_139* | *Prenestra_sp.* |
| *L_54* | *Antaeotricha_sp.* | *L_140* | *sp.24* |
| *L_55* | *Tarema_macarina* | *L_141* | *Carthara_abrupta* |
| *L_56* | *Lurama_sp.* | *L_142* | *Idalus_lineosus* |
| *L_57* | *Stenoma_sp.8* | *L_143* | *Chlamydastis_sp.1* |
| *L_58* | *Heliodinidae_sp.1* | *L_144* | *Megalopyge_braulio* |
| *L_59* | *Anacampsis_sp.* | *L_145* | *Antera_lectabilis* |
| *L_60* | *Symmachia_hippodice* | *L_146* | *Gelechiidae_sp.2* |
| *L_61* | *Dichomeris_sp.6* | *L_147* | *Aristotelia_sp.2* |
| *L_62* | *Inga_sp.3* | *L_148* | *Stenoma_sp.7* |
| *L_63* | *Hemimene_sp.1* | *L_149* | *Stenoma_sp.9* |
| *L_64* | *Dalceridae_sp.1* | *L_150* | *Semyra_incisa* |
| *L_65* | *Ochrotaenia_flexa* | *L_151* | *Compsolechia_sp.5* |
| *L_66* | *sp.14* | *L_152* | *Notodontidae_sp.* |
| *L_67* | *Hylesia_ebalus* | *L_153* | *Stenoma_sp.21* |
| *L_68* | *Episimus_sp.3* | *L_154* | *Dalceridae_sp.* |
| *L_69* | *Dichomeris_sp.1* | *L_155* | *Phycitinae_sp.24* |
| *L_70* | *Compsolechia_sp.7* | *L_156* | *Lacosoma_sp.* |
| *L_71* | *Urodus_sp.* | *L_157* | *Deuterollyta_francesca* |
| *L_72* | *Compsolechia_sp.22* | *L_158* | *Stenoma_salome* |
| *L_73* | *sp.13* | *L_159* | *Stenoma_sp.26* |
| *L_74* | *sp.15* | *L_160* | *Phycitinae_sp.19* |
| *L_75* | *Podalia_annulipes* | *L_161* | *Gelechiidae_sp.28* |
| *L_76* | *Hylesia_sp.1* | *L_162* | *Udramonia_spitzi* |
| *L_77* | *Hesperiidae_sp.2* | *L_163* | *Chlamydastis_sp.* |
| *L_78* | *Thyrididae_sp.1* | *L_164* | *Compsolechia_sp.19* |
| *L_79* | *Stenoma_sp.6* | *L_165* | *Dichomeris_sp.14* |
| *L_80* | *sp.20* | *L_166* | *Dichomeris_sp.4* |
| *L_81* | *Episimus_sp.5* | *L_167* | *Gelechiidae_sp.50* |
| *L_82* | *Antaeotricha_sp.14* | *L_168* | *Gelechiidae_sp.79* |
| *L_83* | *sp.16* | *L_169* | *Phycitinae_sp.22* |
| *L_84* | *Antaeotricha_sp.17* | *L_170* | *Phidotricha_erigens* |
| *L_85* | *Antaeotricha_sp.3* | *L_171* | *Aristotelia_sp.4* |
| *L_86* | *Geometridae_sp.1* | *L_172* | *Scythris_sp.* |
| *L_87* | *Inga_neospila* | *L_173* | *Cyclomia_mopsaria* |
| *L_88* | *Platynota_rostrana* | *L_174* | *Gelechiidae_sp.51* |
| *L_89* | *Stenomatinae_sp.4* | *L_175* | *Ophtamolbysis_eydeira* |
| *L_90* | *sp.17* | *L_176* | *Adelpha_cythrea* |
| *L_91* | *Gelechiidae_sp.10* | *L_177* | *Compsolechia_sp.* |
| *L_92* | *Dalceridae_sp.2* | *L_178* | *Perigea_sp.* |
| *L_93* | *sp.21* | *L_179* | *Hipercompe_sp.1* |
| *L_94* | *Gonioterma_sp.1* | *L_180* | *Hylesia_schuessleri* |
| *L_95* | *Chlamydastis_platyspora* | *L_181* | *Phycitinae_sp.23* |
| *L_96* | *sp.19* | *L_182* | *Gelechiidae_sp.18* |
| *L_97* | *Elachistidae_VOB_840618* | *L_183* | *Dichomeris_sp.3* |
| *L_98* | *Compsolechia_sp.9* | *L_184* | *Cydosia_punctistriga* |
| *L_99* | *Gelechiidae_sp.80* | *L_185* | *Episimus_sp.* |
| *L_100* | *Isognathus_caricae* | *L_186* | *Concana_mundissima* |
| *L_101* | *Gelechiidae_sp.87* | *L_187* | *Praeacrospila_sp.* |
| *L_102* | *Gelechiidae_sp.* | *L_188* | *Dichomeris_sp.9* |
| *L_103* | *Gelechiidae_sp.83* | *L_189* | *Eunica_bechina* |
| *L_104* | *Acraga_ochracea* | *L_190* | *Hypocala_andremona* |
| *L_105* | *Stenoma_sp.1* | *L_191* | *Diaphania_glauculalis* |
| *L_106* | *Stenoma_picta* | *L_192* | *Leuciris_institata* |
| *L_107* | *Stegasta_sp.1* | *L_193* | *Hyperchiria_incisa* |
| *L_108* | *Automeris_illustris* | *L_194* | *Paracles_sp.1* |
| *L_109* | *Phaereoecea_uterella* | *L_195* | *Hesperiidae_sp.* |
| *L_110* | *Cerconota_sciaphilina* | *L_196* | *Oxydia_saturniata* |
| *L_111* | *Parcella_anarynthina* | *L_197* | *Gelechiidae_sp.25* |
| *L_112* | *Hallonympha_paucipuncta* | *L_198* | *Norape_sp.2* |
| *L_113* | *Stenoma_lapilella* | *L_199* | *Chioides_catilus_catilus* |
| *L_114* | *Blastobasidae_sp.5* | *L_200* | *Episimus_sp.2* |
| *L_115* | *Antaeotricha_enodata* | *L_201* | *Ophiolechia_contrasta* |
| *L_116* | *Phycitinae_sp.7* | *L_202* | *Heterocampa_sp.* |
| *L_117* | *Sophista_latifasciata* | *L_203* | *Stenoma_sp.5* |
| *L_118* | *Stenoma_ochropa* | *L_204* | *Hypargyria_definitela* |
| *L_119* | *Blastobasidae_sp.17* | *L_205* | *Gonionota_vivida* |
| *L_206* | *Xeniades_chalestra* | *L_249* | *Gelechiidae_sp.82* |
| *L_207* | *Stenoma_sp.4* | *L_250* | *Gelechiidae_sp.84* |
| *L_208* | *Pseudoplusia_includens* | *L_251* | *Fregela_semiluna* |
| *L_209* | *Aristotelia_sp.7* | *L_252* | *Gelechiidae_sp.86* |
| *L_210* | *Stenoma_sp.11* | *L_253* | *Dichomeris_sp.* |
| *L_211* | *Geometridae_sp.* | *L_254* | *Lethata_sp.2* |
| *L_212* | *Heliodines_sp.2* | *L_255* | *Dalcera_abrasa* |
| *L_213* | *Platyprosterma_perpectinata* | *L_256* | *Megalopyge_lanata* |
| *L_214* | *Phycitinae_sp.6* | *L_257* | *Elbella_azeta* |
| *L_215* | *Norape_sp.* |  |  |
| *L_216* | *Gonioterma_sp.* |  |  |
| *L_217* | *Inga_sp.14* |  |  |
| *L_218* | *Oecophorinae_sp.2* |  |  |
| *L_219* | *Megalopyge_radiata* |  |  |
| *L_220* | *Euphaneta_divisa* |  |  |
| *L_221* | *Stenoma_sp.40* |  |  |
| *L_222* | *Maruca_vitrata* |  |  |
| *L_223* | *Oospila_sp.1* |  |  |
| *L_224* | *Abaera_sp.* |  |  |
| *L_225* | *Syllepte_pactotalis* |  |  |
| *L_226* | *Semyra_sp.* |  |  |
| *L_227* | *Saturniidae_sp.11* |  |  |
| *L_228* | *Dynamine_agacles* |  |  |
| *L_229* | *Stenoma_sp.41* |  |  |
| *L_230* | *Paracles_sp.2* |  |  |
| *L_231* | *Antaeotricha_sp.10* |  |  |
| *L_232* | *Automeris_amoena* |  |  |
| *L_233* | *Tolype_innocens* |  |  |
| *L_234* | *Stenalcidia_sp.3* |  |  |
| *L_235* | *Phycitinae_sp.21* |  |  |
| *L_236* | *Semyra_sp.2* |  |  |
| *L_237* | *Lycaenidae_sp.* |  |  |
| *L_238* | *Miresa_clarissa* |  |  |
| *L_239* | *Lasiothyris_pervicax* |  |  |
| *L_240* | *Strepsicrates_smithiana* |  |  |
| *L_241* | *Chiomara_basigutta* |  |  |
| *L_242* | *Sarrothripinae_sp.1* |  |  |
| *L_243* | *Gelechiidae_sp.85* |  |  |
| *L_244* | *Stenalcidia_sp.* |  |  |
| *L_245* | *Stenoma_sp.10* |  |  |
| *L_246* | *Stenoma_sp.3* |  |  |
| *L_247* | *Oecophorinae_sp.3* |  |  |
| *L_248* | *Eucropia_VOB18747* |  |  |
